# A prognostic analysis of pediatrics central nervous system small cell tumors: evaluation of EGFR family gene amplification and overexpression

**DOI:** 10.1186/1746-1596-9-132

**Published:** 2014-07-01

**Authors:** Weidong Liu, Shigang Zhang, Liyong Zhang, Qingke Cui, Jiyue Wang, Ting Gui, Qi Pang

**Affiliations:** 1 Neurosurgical Department, Liaocheng People’s Hospital, Liaocheng, Shandong Province, China; 2 The Pathology Department, the medical school, Shandong University, Jinan, Shandong Province, China; 3 The Neurosurgical Department, the Affiliated Provincial Hospital, Shandong University, 324# Jingwu Weiqi Road, Jinan, Shandong Province, China

**Keywords:** Medulloblastoma, PNET, Small cell glioblastoma, EGFR, Prognostic analysis

## Abstract

**Background:**

Central nervous system (CNS) tumors are the most common solid tumors that occur in children, however there were few big-data follow-up analysis published in China. Overexpression of epidermal growth factor receptor (EGFR) family members was reported on glioblastoma (GBM) and medulloblastoma (MB) before. However, the correlation between EGFR family members expression with prognosis of MB, supratentorial primitive neuroectodermal tumor (PNET) and small cell GBM is unclear in Chinese children.

**Methods:**

A retrospective and survival analysis was performed on children (age ≤ 16 years) diagnosed as CNS primary small cell tumors in the Affiliated Provincial Hospital, Shandong University from 2000 to 2012, including MB (n = 44), PNET (n = 8) and small cell GBM (n = 19). The expression of EGFR, ERBB-2, ERBB-3 and ERBB-4 were detected by immunohistochemistry (IHC). The fluorescence in situ hybridization (FISH) was used to observe the amplification of EGFR and ERBB-2 gene.

**Results:**

Median survival times of MBs, small GBMs and PNETs were 23 ± 6.7 months, 8 ± 4.7 months and 10 ± 1.4 months. Expression and amplification of ERBB-2, ERBB-3 and ERBB-4 were not observed in all tumor samples. The multiply Cox regression suggested the overexpression and amplification of EGFR were negative prognostic factors for MB. Radiotherapy had the positive function for all pediatric patients.

**Conclusion:**

Overexpression of EGFR predicts poor outcomes of MBs, small cell GBMs and PNETs, suggesting those three CNS tumor subtypes can be considered as one group for the potential common mechanism. The current individual treatment and big data analysis of pediatric CNS embryonal tumors and GBM continues to be very challenging in China.

**Virtual Slides:**

The virtual slide(s) for this article can be found here: http://www.diagnosticpathology.diagnomx.eu/vs/7649640001237474

## Background

Central nervous system (CNS) tumors are the second most common group of malignancies among children; leukemias as a group are the most common. However, CNS tumors are the most common form of solid tumors in children. The overall average annual incidence rate for pediatric CNS tumors (ages 0–19 years) is 5.26 per 100,000 [1]. Embryonal tumors are the most common CNS neoplasms in infants less than 36 months of age and are described by the World Health Organization (WHO) classification scheme as undifferentiated small round cell tumors with divergent patterns of differentiation [2]. Embryonal Tumors include primitive neuroectodermal tumor (PNET), medulloblastoma (MB), atypical teratoid/rhabdoid tumor, and several other histology types. Though those tumors in this category are histologically similar, they have different patterns of incidence and survival, so it is important to look at them individually.

In USA (United States of America), the incident rate of CNS embryonal tumors under 14 years old is 0.8 per 100,000 and the median age is 9. In the age 0 ~ 4, the embryonal tumors are the most common histology, and between age 5 ~ 14, the embryonal tumors are still the third most common subtype compared with pilocytic astrocytoma [1]. Conversely, there were few reports related with the large-scale follow-up or prognostic analysis of pediatrics CNS embryonal tumors in china.

MB comprises up to 20% of all pediatric brain tumors and is currently treated with surgical resection, radiation therapy, and chemotherapy [3]. Molecular genetic parameters, being associated with poorer prognosis of MB, include overexpressed ERBB-2, high MYCC expression, and possibly p53 accumulation [4]. The single most-predictive clinical factor is extent of disease at the time of diagnosis, patients with disseminated disease fare less well [5]. Especially ERBB-2, belonging to the human epidermal growth factor receptor (EGFR) family, is overexpressed in 40% of MBs and its expression correlates with poor outcome [6]. However, ERBB-2 expression in PNET is unclear and should be invested. PNET is histologically similar to classic MB and constitutes 2% of all childhood brain tumors. The most common sites of PNET onset are the cerebrum, suprasellar, or pineal region of children in their first decade of life [6]. Because MB and PNET share the aggressive biological behavior, it is crucial to determine the prognostic factors for guiding the individual treatment.

Glioblastoma multiforme (GBM) is the second most frequently reported malignancy in CNS, which account for 15.6% of all primary brain tumors in adults. GBMs are more common in older adults and are uncommon in children [1]. GBM is frustratingly chemoresistant and follows a highly aggressive course, with an average survival of roughly 1 year. Although small cells are common in GBM, they are predominant or exclusive in a subset known as small cell GBM [7]. Small cell GBM is a histological subtype of GBM with characteristic features of highly proliferative, monotonous small glial cells with high nuclear cytoplasm ratio. In this study, we also focused on the prognostic research for small cell astrocytoma/GBM for the reason that it shares some similar features with embryonal tumors. The cytogenetical investigations for IDH1/2 mutation, 1p/19q loss, and PTEN alteration are strongly supportive methods for the differential diagnosis of small cell GBM [8]. PTEN also represents a putative tumor suppressor gene in MB because loss of PTEN function would contribute to an over-activation of the PI3K/AKT signaling pathway, which is activated in MB [9]. Mutations in the IDH1/2 genes are similarly detected in patients with non-glial tumors with the exception of PNET, which suggesting the unique mechanism of PNET shared with small cell GBM [10]. Therefore, small cell GBM shares the similar genetics and histopathology features with MB and PNET, and we group them together as pediatrics CNS small cell tumors in this study.

The members of the EGFR family have been linked to the astrocytic tumors malignant transformation. This receptor family consists of four tyrosine kinase receptors, ERBB1-4, and seems to be involved in tumor cell proliferation, differentiation and cell survival [11]. Due to overexpression of the ERBB1-4 proteins on the surface of neoplastic astrocytes, they are candidates for targeted therapy [12]. Such treatment, however, requires reliable detection systems for these receptor proteins in tumor tissue. EGFR gene amplification can now simply be evaluated by means of fluorescence in situ hybridization (FISH) [13]. Several studies have shown a varying degree amplification of the EGFR (ERBB1) gene, located on chromosome 7, in GBM [14]. EGFR gene amplification distinguishes small cell GBM from anaplastic oligodendrogliomas [7], nevertheless the spectrum of clinicopathologic and prognostic features has not been explored fully in small cell GBM. And the other members of EGFR family expression levels and their correlation with prognosis are also unclear.

In china, according our investigation, most hospitals even only offer patient surgical resection without radiation or chemotherapy. It is important to raise the neurosurgeons and oncologic doctors attention for combining various forms treatment to increase young patients disease-free survival time and improve the quality of life. For pathologists’ responsibility, finding the prognostic factors of pediatrics CNS small cell tumors becomes the important task to give the physicians suggestion. This study was also designed to investigate the clinical features and the extent of ERBB-1 ~ 4 gene expression in the small cell GBM, PNET and MB. Further and most importantly, we assumed to explore the prognosis factors in children small cell CNS tumors.

## Data collection and methods

### Clinical data collection

All 71 pediatric (≤16 year-old) CNS primary small cell tumors out of 383 children CNS primary tumors (18.54%) were operated at the Department of Neurosurgery, Affiliated Provincial Hospital, Shandong University, Jinan, China, and consecutively collected in the time period 2000 to 2012, including 44 cases medulloblastoma (MB), 8 cases primitive neuroectodermal tumor (PNET) and 19 cases small cell glioblastoma (GBM). A statistical analysis was performed to collect demographic and clinical data that included age, sex, tumor localization, treatment modalities, and postoperative survival (Additional file 1).

Craniotomies were performed under general anesthesia, and all patients underwent magnetic resonance imaging (MRI) a few days before and within 72 hours after surgery. The extent of tumor resection was determined by the postoperative MRI scans, defined as gross total resection or partial resection (residual volume exceeding 2 cm). The data and tumor specimens were retrieved and revised in 2013 by neurosurgeons and pathologists in Provincial Hospital according to the 2007 WHO Classification of Tumors of the Central Nervous System [2]. All specimens were taken during the patients’ first surgery.

### Immunohistochemistry (IHC)

Expression of ERBB1-4 receptor proteins was determined by IHC using commercial monoclonal antibodies EGFR (1:50, Santa Cruz), ERBB-2 (1:50, Santa Cruz), ERBB-3 (1:50, Santa Cruz) and ERBB-4 (1:50, Santa Cruz). The proliferation of tumor cells was detected by ki-67 IHC staining. Formalin-fixed and paraffin-embedded sections, 4 μm thick, with representative tumor tissue, were incubated with primary antibodies after antigen retrieval by pressure autoclaving. An automatized histostainer was used for the immunohistochemcial procedures (Dako Autostainer, Denmark). For visualization of immunoreactivity, DAKO EnVision system was used with diaminobenzidin as chromogene. Sections were counterstained with haematoxylin. Positive controls were included in each staining run.

The immunoreactivity was assessed by means of intensity and percentage of immunoreactive tumor cells. Intensity was recorded as 0 (no reaction) to 3 (strong reaction). Fraction of immunoreactive tumor cells was recorded as 0 (no positive cells), 1 (<10% positive cells), 2 (10-50% positive cells), or 3 (>50% positive cells). A staining index was calculated as the product of intensity and fraction of positive tumor cells [15].

### FISH (Fluorescence in situ hybridization)

The copy number of EGFR and ERBB-2 can be determined by direct fluorescence in situ hybridization (FISH) [16,17].

EGFR/CEN-7 FISH Probe Mix (DakoCytomation) and the Histology FISH Accessory Kit (Dako) were applied for gene copy number detection of the EGFR gene located on chromosome 7p11.2 and for copy number detection of the chromosome 7 centromere region (chromosome 7 copy number detection). The Red-labeled DNA probe (EGFR) binds to a 196 kb segment containing the EGFR gene on chromosome 7q11.2. The fluorescein Green-labeled probe (CEN-7) binds to the centromeric region of chromosome 7. The PathVysion ERBB-2/HER-2 probe kit (Abbott Molecular) was used for the FISH analysis. The protocol was similar with EGFR gene FISH staining. Slides were hybridized with prewarmed probes for the ERBB-2 gene (orange) and chromosome 17 centromere (Her2/neu/CEP17 SG probe, Vysis) overnight at 37°C.

In brief, the sections were de-paraffinised using xylene, rehydrated, and pretreated using DAKO solution kit (Histology FISH Accessory kit). The probes were added to the sections, coverslipped, sealed with rubber cement, and placed in a DAKO Hybridizer. The sections and probes were co-denatured for 5 min at 73°C, followed by annealing at 37°C over night. After hybridization slides were washed in 0.4 × SSC (with detergent) at 73°C for two minutes followed by one minutes in 2 × SSC at room temperature. Then the sections underwent dehydrating in ethanol three times for 3 min. The slides were counterstained and embedded with a 4,6-diamidino-2-phenylindole/antifade solution (DAKO). FISH signals for each locus-specific FISH probe were assessed under a Nikon Eclipse 90i microscope (Nikon, Tokyo, Japan) equipped with a triple-pass filter (DAPI/Green/Orange).

The signal enumeration was performed under high magnification (×1,000). The entire area of tumor tissue was evaluated in each case, and all nuclei were assessed for the orange (marker) and green (reference) signals. For a patient to be included, 100 evaluable cells were to be assessed. Tumors were interpreted as amplified when the ratio of HER-2/EGFR signals divided by chromosome 17/7 centromere signals was equal to or greater than 2.2 and the normal specimens showed a ratio of <1.8. Results at or near the cutoff point (1.8 – 2.2) were repeated with a fresh specimen slide.

### Statistics and follow-up analysis

Statistical analyses were made using SPSS version 16.0 (SPSS Inc., Chicago, IL). Survival time was calculated from date of surgery to date of death or the termination of observation. Multiple Cox regression analyze was used to study the association between sex (categorical variable; male versus female), age (continuous variable), tumor size (continuous variable), extent of surgical resection (categorical variable; gross total resection versus partial resection), the treatment (categorical variable; no radiotherapy versus radiotherapy), ki-67 (Staining Index, continuous variable), ERBB-1 ~ 4 protein expression (Staining Index, continuous variable) and survival prognosis. The Kaplan-Meier method was applied to draw the survival curves and the log-rank test was used for survival analysis. The association between results from the FISH investigations (categorical variables; positive versus negative) and survival were studied in the same manner. The relationship of IHC and FISH staining between the different tumor groups was analyzed by non-parametric Kruskal-Wallis H method. Two-sided P-values less than 0.05 were regarded as statistically significant.

The study was approved by the Committee for Medical Research Ethics of Affiliated Provincial Hospital, Shandong University.

## Results

### The clinical features and survival times of pediatrics CNS small cell tumors

**Figure 1:**
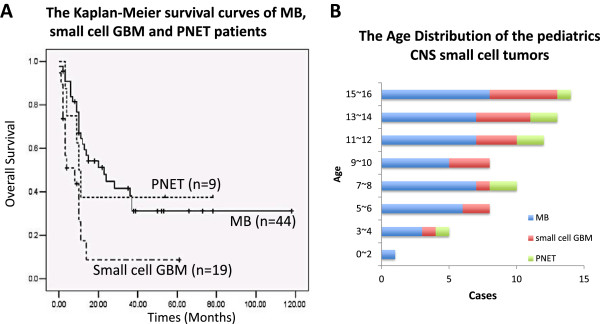
**The age distribution and keplan-meier survival curves of the pediatrics (≤year 16) CNS small cell tumors. A)** The age distribution of the pediatrics CNS small cell tumors including 44 cases medulloblastomas (MB), 8 cases PNETs and 19 cases small cell glioblastoma (GBM) diagnosed in the Affiliated Provincial Hospital, Shandong University from 2000 to 2012. **B)** The keplan-meier survival curves of MB, small cell GBM and PNET pediatric patients. P values: MB: small cell GBM, 0.0005; MB: PNET, 0.0005; small cell GBM: PNET, 0.12. P-values were obtained from two-sided log-rank tests.

From January 2000 to December 2012, 71 cases pediatric (≤16 year-old) CNS primary small cell tumors were followed up including MB (44 cases), small cell GBM (19 cases) and PNET (8 cases). At the end of December 31, 2012, 65 patients were interviewed, of which 40 MB patients, 17 cases small cell GBM and 8 cases PNET, accounted for 91.55% of total small cell tumors. 45 patients were dead at the endpoint of observation. The follow-up time was 4 months to 156 months, with an average time of 84 months. The median survival time of MB (23 ± 6.7 months) was much longer than small cell GBM (8 ± 4.7 months) and PNET patients (10 ± 1.4 months), respectively (Figure 1A).

In 44 cases MB, 27 males and 17 females (M: F =1.59:1) were involved and the median survival time of female was 24 months while 15 months for men. Of 8 cases PNET, 4 males and 4 females were calculated and the median survival time of female (10 months) was similar with male (9 months). Because of the restriction of patient number, statistics was not applied for PNET. In 19 cases small cell GBM, 13 males and 6 females were included, and the median survival time of female was 10 months comparing with 6 months of male. Although CNS small cell tumors were more common in male and the median survival time is longer for female, there was no significant difference between genders (P > 0.05) (Tables 1, 2, 3).Compared with small cell GBM and PNET, the age of onset in MB was younger (year 1.8 ~ 16) and the peak age was year 8 to 12. The average ages of onset were 11 and 10.5 in the small cell GBM and PNET respectively, and especially for GBM, the number of patients was increased with the growth of age (Figure 1B).

**Table 1:** The clinicopathology features and survival time distribution of MB patients

**MB**	**Number of patients**	**Median survival times**	**P value**
44 cases	(Death)	X ± s (months)	(<0.05)
**Sex**			0.346
Male	27(19)	15 ± 5.769	
Female	17(12)	24 ± 11.406	
**Resection stage**			
Total resection	33(17)	35 ± 4.763	0.0352
Partial resection	11(9)	20 ± 6.020	
**Therapy**			<0.0001
Surgery only	27(23)	10 ± 0.975	
Surgery + Radiotherapy	17(3)	53 ± 10.29	
**Ki67**			0.051
0,1+, 2+	21(8)	51 ± 10.29	
3+	13(8)	20 ± 5.503	
**EGFR (IHC) (34 cases)**			0.0001
0,1+, 2+	20(5)	53 ± 20.82	
3+	14(11)	11 ± 3.338	
**EGFR (FISH) (34 cases)**			0.002
Negative	15(5)	53 ± 19.96	
Positive	19(11)	20 ± 7.028	

**Table 2:** The clinicopathology features and survival distribution of small cell GBM patients

**GBM**	**Number of patients**	**Median survival times**	**P value**
19 cases	(Cases were dead)	X ± s(months)	(<0.05)
**Sex**			0.365
Male	13(10)	8 ± 4.676	
Female	6(4)	10 ± 0.031	
**Resection stage**			0.0744
Total resection	11(8)	10 ± 1.309	
Partial resection	8(6)	4 ± 3.771	
**Therapy**			0.00031
Surgery only	12(10)	3 ± 0.476	
Surgery + Radiotherapy	7(4)	11 ± 1.095	
**Ki67**			0.0359
0,1+	9(6)	10 ± 2.449	
2+, 3+	6(4)	4 ± 0.376	
**EGFR (IHC) (15 cases)**			0.0085
0,1+, 2+	7(5)	11 ± 1.138	
3+	8(5)	3 ± 1.194	
**EGFR (FISH) (15 cases)**			0.0267
Negative	8(5)	11 ± 1.646	
Positive	7(5)	3 ± 1.047	

**Table 3:** The clinicopathology features and survival distribution of PNET patients

**PNET**	**Number of patients**	**Median survival times**
8 cases	(Cases were dead)	X ± s (months)
**Sex**		
Male	4(3)	9 ± 4
Female	4(2)	10
**Resection stage**		
Total resection	4(1)	54
Partial resection	4(4)	10 ± 1.414
**Therapy**		
Surgery only	4(4)	4 ± 3
Surgery + Radiotherapy	4(1)	43 ± 1.047
**Ki67**		
0,1+	6(3)	
2+, 3+	2(2)	
**EGFR (IHC) (8 cases)**		
0,1+, 2+	4(1)	
3+	4(4)	4 ± 0.816
**EGFR (FISH) (8 cases)**		
Negative	4(1)	
Positive	4(4)	4 ± 0.816

The most common site of MB was cerebellar vermis (30/44) and the average volume was 4.92 cm. The lesion site of small cell GBM was mainly observed in the cerebral hemispheres (12/19), among which temporal lobe (6/19) was most usual onset location and the average volume was 5.13 cm. While half of PNET occurred in the cerebral hemispheres (4/8), the average volume was 5.25 cm.

**Figure 2:**
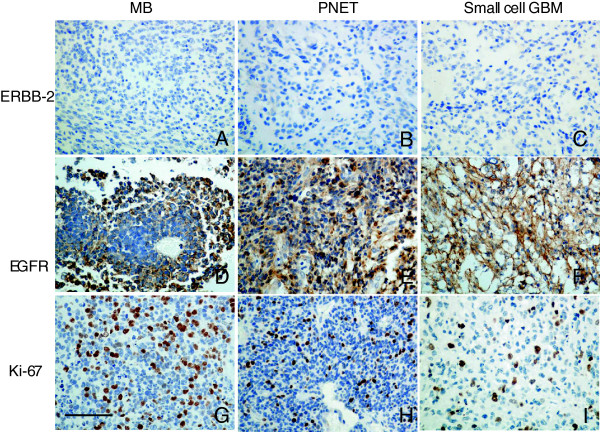
**The immunohistochemistry staining of ERBB-2, EGFR and ki-67 in MB, PNET and small cell GBM. A ~ C)** The ERBB-2 expression in MB, PNET and small cell GBM respectively; **D ~ F)** The EGFR expression in MB, PNET and small cell GBM respectively (The staining index: 3+); **G ~ I)** The ki-67 expression in MB, PNET and small cell GBM respectively (The staining index: 3+). Original magnification × 400. The bar: 50 μm.

Ki-67 staining was applied for the tumor proliferation determination, and there was no significant difference of ki-67 expression between 3 different tumor groups (P = 0.305). The positive cases (index 1+, 2+ and 3+) were accounted for 94.12%, 66.67% and 85.71% of MB, small cell GBM and PNET respectively (Figure 2 Ki-67 staining). The median survival time was presented increasing tendency when the percentage of positive ki-67 staining was lower than 50% in these 3 tumor subtypes, although only small cell GBM patients showed statistical difference (Tables 1, 2, 3).

### The EGFR gene amplification and protein expression in pediatric CNS small cell tumors

Tumor tissue samples were available from 34 of the 44 patients with MB and 15 out of the 19 patients with small cell GBM. Those tissue samples and all PNET samples were evaluable by IHC and FISH, although PNET group did not meet the criteria for statistical analysis because of too many censored values. The histologic features were similar for MB, PNET and small cell GBM. Microscopically, sheets and compact nests of uniform small blue cells with scant cytoplasm were seen in all lesions, however the well-formed rosettes were lavish in MB and necrosis was more common in small cell GBM (Figure 2).

**Figure 3:**
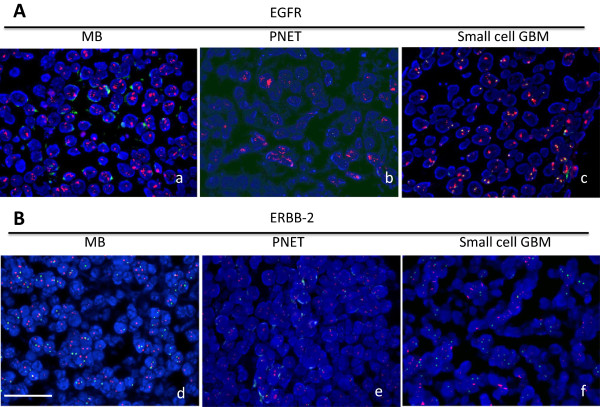
**The Fluorescence in situ hybridization of EGFR and ERBB-2 in MB, PNET and small cell GBM. A)** The positive signals of EGFR gene amplification in MB **(a)**, PNET **(b)** and small cell GBM **(c)**; **B)** The signals of EGFR gene in MB **(d)**, PNET **(e)** and small cell GBM **(f)**. The bar: 25 μm.

The positive IHC expression of EGFR cases accounted for 91.18%, 86.67% and 71.43% of MBs, small cell GBMs and PNETs respectively (Figure 2), and there was no statistical different between 3 tumor subtypes (P = 0.0636). The negative expression (0) and weakly positive expression (1+, 2+) cases were consolidated into the low expression group, compared with strong positive cases (3+). The prognosis of all tumor subgroups with EGFR IHC low expression (0 ~ 2+) was promising than the one of high expression groups (3+) by log-rank analysis (Tables 1, 2, 3).Dual-color FISH analysis was carried out to evaluate for the gene amplification in the tumor lesions by the number of signals in each cell related to EGFR and to the centromeric region of chromosome 7. Representative positive images are illustrated in Figure 3A. The average ratio of EGFR/centromeric region of human chromosome 7 signals was more than 2.0 as calculated positive signals in each tumor group. The cells with orange clusters were also determined as positive expression.

The EGFR gene was amplified in 19 out of 34 MB tumors (55.88%), 7 from 15 small cell GBM cases (46.67%) and 4 cases of 8 PNET samples (50%) assessed by FISH (Figure 3A). In the vast majority of specimens, the amplification was widespread, typically involving nearly all tumor cells. However, 2 PNET specimens had focal EGFR amplification, with only 5–10% of tumor cells identified as positive. The log-rank survival analysis showed that all CNS small cell tumors patients with EGFR FISH positive signals had longer median survival time compared with ones with negative signals (P ≈ 0.002 for MB and GBM) (Tables 1, 2, 3).

We also analyze the relationship between EGFR IHC and FISH staining in 3 subgroups and found that the patients with both protein high expression and gene amplification suffered from the worst prognosis compared with ones with double negative signals (Table 4). The overall concordances between EGFR gene amplification status by FISH and EGFR IHC were 61.76% of MB, 75% of PNET and 80% of small cell GBM. Notably, EGFR IHC negative samples should also apply the FISH examination to help analyze the patient prognosis, for the reason that the patients who was detected with negative EGFR protein expression but gene amplification also suffered from poor prognosis.

**Table 4:** The FISH and IHC detection of EGFR signals in CNS small cell tumors

	**CNS small cell tumors**	**The number of patients (median survival time)**	
		**FISH**	
IHC	MB (34)	+	-
	+	10 (10.75 ± 4.1)	4 (29.51 ± 2.33)
	-	9 (21.14 ± 3.29)	11 (59.18 ± 10.21)
		FISH	
	GBM (15)	+	-
	+	6 (2.5 ± 1.1)	2 (4)
	-	1 (10)	6 (12.27 ± 2.21)
		FISH	
	PNET (8)	+	-
	+	3 (3.5 ± 2.1)	1 (11)
	-	1 (5)	3 (all alive > 54)

Note: FISH: Fluorescence of in situ hybridization; IHC: immunohistochemistry.

### The ERBB-2, 3 and 4 gene amplification and protein expression in CNS small cell tumors

Although previous research suggested that ERBB-2 was considered as the prognosis factors for the MB [3]. We did not observe the clear IHC positive signals of ERBB-2 in MB, small cell GBM and PNET samples (Figure 2 ERBB-2). To determine the ERBB-2 gene amplification, FISH was applied in 10 cases MBs, 10 cases small cell GBMs and 4 cases PNETs samples. The results overlapped with protein expression, no positive signals observed (Figure 3B). The ERBB-3 and 4 IHC expression was also negative in all CNS small cell tumor samples (Data not shown because of the similar features with ERBB-2).

### The children CNS small cell tumors treatment options and their impact on prognosis

Patients with small cell GBM faced a poor prognosis regardless of surgical procedures, while MB patients with partial resection suffered worse prognosis compared ones with total resection (P < 0.0352) (Tables 1 and 2). Although the statistic between two surgical resections was not calculated because of restriction of patient number for PNET, the survival time of patients with total resection was more than 45 months at from time of onset to the follow-up endpoint (Table 3). The total resection had the positive impact for the prognosis of MB and PNET patients.

The study did not involve the radiation exposure time and dosage, and the result was only the survival function of postoperative radiotherapy for the prognosis. The total 28 CNS small cell tumors patients (39.44%) were received postoperative radiotherapy,among which 38.64% (17/44) MB patients, 50% (4/8) PNET patients and 36.84% (7/19) small cell GBM respectively. Statistical analysis showed that radiotherapy had positive function for every group tumors (P <0.01) with regardless of surgical resection (Tables 1, 2, 3). The median survival time of MB, PNETs and small cell GBMs underwent radiotherapy was longer compared with the pediatric patients only received surgical resections. To be noticed, the majority of those received radiotherapy patients were from the urban population, while patients without undergoing radiotherapy and suffered from larger tumor volumes during surgeries were mainly from economically underdeveloped rural areas (Data not shown).

### The multiple Cox regression analysis of pediatrics CNS small cell tumors

The multiple Cox regression analysis with overall survival as the dependent variable was performed for CNS small cell tumor clinical features with expression of EGFR protein expression and gene amplification (Tables 5, 6, 7). After adjusting for age, gender, tumor size, tumor invasion, ki-67, extent of surgical resection and treatment, the Cox analysis revealed that without-radiotherapy, EGFR protein overexpression (IHC) and EGFR gene amplification (FISH) were the statistically significant poor prognostic factors in patients with MB (P = 0.000013). The other clinical features, tumor invasion and ki-67 reached no significance. The small cell GBM and PNET shared the same poor prognosis factors which were without-radiotherapy and EGFR protein overexpression. The limited number of tumor cases may be the reason for the EGFR amplification was not involved in the Cox regression of small cell GBM and PNET.

**Table 5:** The multiple cox regression analyses of MB

**The prognosis related factors**	**RR (relative ratio)**	**P**
Radio-therapy	27.81616515	0.0000133
EGFR (IHC)	14.31869361	0.000154325
EGFR (FISH)	8.9986669	0.002701766
**No-related factors**	**Score**	**P**
Age	4.021743642	0.054917253
Gender	0.060643558	0.805481647
Tumor size	1.300125433	0.254190313
Resection extent	2.503056978	0.113625548
Ki-67	3.761288415	0.052452147
Invasion	0.841301195	0.359024894

**Table 6:** The multiple cox regression analyses of small cell GBM

**The prognosis related factors**	**RR**	**P**
Radio-therapy	9.85	0.0017
EGFR (IHC)	8.34	0.00389
**No-related factors**	**Score**	**P**
Age	0.00762	0.93
Tumor size	0.002	0.97
Resection extent	0.009	0.92
Ki-67	1.302	0.25
EGFR (FISH)	1.526	0.22
Vascular proliferation	2.162	0.14

**Table 7:** The multiple cox regression analyses of PNET

**The prognosis related factors**	**RR**	**P**
Radio-therapy	6.624	0.01
EGFR (IHC)	5.66	0.022
**No-related factors**	**Score**	**P**
EGFR (FISH)	4.82	0.038
Age	0.697057605	0.403774151
Gender	0.999869375	0.317342117
Tumor size	2.789838274	0.094863801
Resection extent	2.190441949	0.138869465
Ki-67	3.55940479	0.059208966
Invasion	1.772277228	0.183100505

## Discussion

In this research, we concentrated on the prognostic factors for the pediatrics CNS small cell tumors contained small cell GBM, PNET and MB. In the Background part, we already indicated the reason why we grouped them together for the similar genetics or histopathology characters. EGFR protein overexpression was determined as the negative prognostic factor for all small cell CNS tumors in our research. EGFR gene amplification was evaluated necessarily by means of FISH in MB. Even for the small cell GBM and PNET, the disease-free survival time of negative FISH signals was longer than the one of positive signals. We assumed that the limited number of cases was the reason for that the Cox analyses excluded the EGFR amplification from the prognostic factors of small cell GBM and PNET.

The relationship has been correlated the presence of small cell architecture in primary GBM with EGFR amplification [18]. Combination of an anti-EGFR agent Iressa and a JAK2/STAT3 inhibitor synergistically suppressed STAT3 activation and potently killed GBM cell lines that expressed EGFR [19]. However, Alterations of CDKN2A, EGFR, CDK4, and MDM2 genes, commonly implicated in gliomagenesis, were not identified in any PNET, which was opposite with our results [20]. Aberrant activation of Hedgehog (HH) signaling has been identified as a key etiologic factor in MB [21]. Frank Götschel et al. identified a novel crosstalk mechanism whereby EGFR signaling silences proteins acting as negative regulators of HH signaling [22]. The effects of the EGFR inhibitor gefitinib on cell growth and signaling were evaluated in three MB cell lines (D283, D341, Daoy), and gefitinib induced G0/G1 arrest in all lines, indicating that gefitinib might be a molecularly targeted agent for the treatment of MB [23]. As those data indicated, the mechanism of EGFR over-activation in CNS small cell tumors is still ambiguous. The deepen study combining clinical bed to bench is required. At the meantime, the molecular inhibitors of EGFR tyrosine kinases (erlotinib or gefitinib) should be considered as the effective individualized treatment and start the consortium study in China for pediatrics CNS small cell tumors.

According the previous results, ERBB-2 and ERBB-3 mRNAs were detected only in a few high-grade gliomas, while ERBB-4 expression was most pronounced in low-grade gliomas [24]. It was also reported that ERBB-2 and ERBB-4 are highly expressed in aggressive forms of medulloblastoma [25]. However, ERBB-4 expression was downregulated in HH signaling-induced MBs from mice. According to the animal experiments, HH signalling inhibited ErbB-4 expression in mouse cerebellar granule progenitors and human MB cells [26]. And the other researchers also suggested that expression of ERBB-2 is much lower on MB tumor cells than on breast cancer cells, so they are not susceptible to ERBB-2 monoclonal antibodies, like trastuzumab (Herceptin) [5]. The expression and amplification of ERBB-2 and ERBB-4 were not pronounced in MB, PNET and small cell GBM detected by FISH and IHC according our results. Expanding the number of cases will offer more evidence for the ERBB-2 and ERBB-4 expression. It is now recognized that MB is a collection of heterogeneous entities with disparate demographics, transcriptomes, genetics, and clinical outcomes [27]. According to international consensus, the principle subgroups of MB are WNT, SHH, Group 3, and Group 4 [28]. Because our prognostic study did not account for these subgroups, we hypothesized that the difference of EGFR family members expression in MB could have resulted from differential subgroup representation among studies.

We have found that all children patients involved in this study did not receive any chemotherapy and only 39.44% of patients underwent the radiotherapy after surgery. Other researchers have reported a median survival of 14.3 months for small cell GBM [29]. With present means of surgery, craniospinal radiotherapy, and chemotherapy, between 75% and 90% of children greater than 3 years of age with nondisseminated MB are likely to be survivors 5 years after treatment [4]. Compared with those promising prolonged survival time in western countries, the median time of MB and small cell GBM in our hospital was only 2 years and 8 months. We assumed that the short disease-free survival time was affected by the less of radiotherapy. Infants and children with supratentorial PNET and MB are special compared with adult patients for long-term neurocognitive development being considered. This is not only due to the whole-brain radiation therapy these children are often treated with, but also due to the local effects of the tumor and the need for higher-dose boost radiotherapy. In the future, the children cognitive levels should be involved when considering the treatment and prognosis, which will lead more challenge for neurosurgeons and social work.

## Conclusion

Overexpression of EGFR predicts poor outcomes of MBs, small cell GBMs and PNETs, suggesting those three subtypes can be considered as one group for the potential common mechanism. The current individual treatment and big data analysis of pediatric CNS embryonal tumors and GBM continues to be very challenging in China. Our data provided information for the planning of pediatric CNS tumor treatment and control programs, especially for prognosis prediction and the necessary of post-surgery radiotherapy. The more detailed and large-scale follow-up analysis is summoned in China.

## Consent

Written informed consent was obtained from the patient’s parent for the publication of this report and any accompanying images.

## Abbreviations

CNS: Central nervous system; MB: Medulloblastoma; PNET: Primitive neuroectodermal tumor; GBM: Glioblastoma multiforme; EGFR: Epidermal growth factor receptor; FISH: Fluorescence in situ hybridization; IHC: Immunohistochemistry.

## Competing interests

The authors declare that they have no competing interests.

## Authors’ contributions

WL and QP made substantial contributions to conception and design, and analysis and interpretation of data; WL, TG and LZ carried out the immunohistochemistry. QC and LZ participated in the FISH staining. WL, SZ, LZ, QC, JW and TG participated in the design of the study and performed the statistical analysis. WL, SZ and JW were involved in drafting the manuscript or revising it critically for important intellectual content; QP have given final approval of the version to be published; and WL and QP agree to be accountable for all aspects of the work in ensuring that questions related to the accuracy or integrity of any part of the work are appropriately investigated and resolved. All authors read and approved the final manuscript.

## Supplementary Material

Additional file 1 The clinical information of central nervous system small cell tumors pediatric patients. Click here for file
